# Stress and strain among merchant seafarers differs across the three voyage episodes of port stay, river passage and sea passage

**DOI:** 10.1371/journal.pone.0217904

**Published:** 2019-06-04

**Authors:** Marcus Oldenburg, Hans-Joachim Jensen

**Affiliations:** Institute for Occupational and Maritime Medicine Hamburg (ZfAM), University Medical Center Hamburg-Eppendorf (UKE), Hamburg, Germany; Universita degli Studi di Verona, ITALY

## Abstract

**Background:**

A sea voyage is characterized by a variety of work requirements for the ship's crew, basically reflected in three voyage episodes: port stay, river passage and sea passage. The primary aim of this study was to compare stress and strain amongst a sample of merchant seafarers across these three voyage episodes.

**Methods:**

In a cross-sectional maritime field study, 323 sailors on 22 container ships were biometrically surveyed and completed a questionnaire. In addition, a survey of energy expenditure and heart rate (variability) was carried out in parallel with 236 participants with the SenseWear armband monitor and the RS 800 polar watch.

**Results:**

Port stay and sea passage each accounted for the largest proportion of the ships’ journeys, each at around 40%. The study participants rated port stay with 37.8% as the voyage episode with the highest strain, followed by the river passage (24.8%) and then the sea passage (13.0%). The working time during the sea passage was on average shorter than during port stay or the river passage (p<0.001)—as a result, seafarers had more spare time to spend on leisure and sleep. Total energy turnover and, by trend, work energy turnover were notably at the lowest during the sea passage. In particular, the crew had a significantly lower heart rate during the sea passage than during the other two voyage episodes (p = 0.001). Furthermore, there was no difference in the seafarers’ heart rate variability between the voyage episodes.

**Conclusion:**

In the present study, it becomes clear that an accumulation of psychophysical stress takes place during port stay and leads to a subjectively and objectively higher strain level. In contrast, seafarers are more likely to recover during the sea passage. This knowledge should be used to offer ships' crews targeted health measures, in particular during the sea passage.

## Introduction

It has repeatedly been described that ships’ crews are exposed to high levels of psycho-physical stress during their time on board, which usually lasts for several months [1–4]. These burdens result from psychosocial factors, for example, the long-term separation from the family, friends, and other social structures (such as parish, sports club) on land [5, 6]. In principle, these contacts represent an important resource for reducing and compensating for work-related stress when there is a possibility for immediate communication [7–9]. The possibilities for home-based communication, which are limited on board, are often restricted to certain places (only in the next port) and limited in time (during the short stays in port) and thus cannot adequately satisfy the communication needs of many seafarers [10]. Due to the usually multicultural ships’ crews today, language difficulties often present further communication barriers on board, which can lead to isolation and loneliness of crew members [11].

On board, further burdens often result from irregular, often extended working hours, which occur in particular on small ships used near shore and with a high port frequency [12]. The continuous exposure to physical stress due to noise, vibration, ships’ movements over a period of 24 hours (during free time and working hours) and for 9 months in a row, represent additional burdens on seafarers that are not found in land-based working environments [13]. Furthermore, the inequities experienced by seafarers from low- and middle-income countries can also contribute to mental stress, including the linking of nationality to senior positions, longer tours of duty, and different pay rates for the same job [14].The maritime industry has changed in recent decades. The growing rationalization and economization of ship consignments with consequently reduced shore leave and steadily increasing ship dimensions will continue. Today, crew numbers are decreasing while there is a significant increase in security and administrative burdens, especially during port stay [15]. Therefore, it must be assumed that demands placed on the crew of the ship will be in particular during this voyage episode [16, 17].

Due to the high level of effort needed to organize maritime field studies, there are hardly any such studies available to date that assess the stress burden of ships’ crews under the extremely demanding working conditions on merchant vessels [1, 2]. The primary aim of this study was to compare stress and strain amongst a sample of merchant seafarers across the three voyage episodes of port stay, river passage, and sea passage.

## Methods

### Participants and procedures

As part of a maritime field study, two to four scientists conducted psychosocial and biometric surveys on 22 sea voyages of 22 container ships under German management. The study spanned almost 180 days of investigation on board. The examined ships operated in the North Sea area (including the English Channel) or in a similar coastal operation with a high port frequency. The workloads and stress during the journeys were distinguished in the following three voyage episodes:

1. Port stay: after docking at the terminal until the departure begins
2. River passage: the distance from leaving the anchorage (including mooring and take-off maneuvers) to reaching the open sea. The cruise is usually initiated with the inclusion of a district or channel pilot on board for advice on navigation with regard to the respective port and area specifics. Depending on the water area, the river passage can also correspond to a canal trip.
3. Sea passage: voyage of the ship on the open sea, during which no pilot is present on board.

As the responsible nautical officer on board the examined ships logged the exact time of both the arrival and departure as well as the pilot’s boarding or disembarking in the ship's journal, the voyage episodes could be determined to the minute with the help of these records. The episodes of the voyages were continuously recorded according to the entries in the ship’s journal.

The crews of 22 ships from 12 different shipping companies were included (5 ships of company 1 participated, 3 ships of company 2, 2 ships of company 3–6 and 1 ship of company 7–12). Of the total of 365 seafarers, 323 exclusively male sailors took part in this survey (participation rate 88.5%). The mean age was 38.2 (SD 11.8) years. The participation in the study was completely voluntary and the data collection was pseudonymized. All participants gave their written informed consent before taking part in this study. The study was approved by the Ethics Committee of the Hamburg Medical Association (no PV4395).

### Study materials

The sailors were initially asked which of the three voyage episodes they found to be generally the most stressful. In accordance with the International Organization for Standardization (ISO) 10075–1:2017 [18], this study defined stress as the entirety of measurable external influences. Furthermore, the seafarers on board were requested to subjectively assess their experienced physical and mental strain as a result of the activities carried out in the respective voyage episodes. Thus, strain was defined as an effect of stress. Every sailor evaluated each of the three voyage episodes at least once during the sea voyage, providing the information as soon as possible after completion of the respective voyage episode.

All questions used were derived from an own previously published study on seafaring stressors [3]. They were developed for a multicultural crew with different English-speaking and educational backgrounds. According to the preliminary investigations [3], a total of 7 items were included in the questionnaire (6 items had to be answered on a 3-point scale and 1 open-ended item). The 3-point scale items comprised their subjective experience regarding physical and mental strain and strain caused by noise, vibration, heat and ships’ movement (S1 Questionnaire). The items ranged from 'not at all strainful' to 'very strainful', which enabled the crew members to easily distinguish between the levels of strain perception. In order to facilitate the understanding of the medically correct wording “strainful”, this term was described in the questionnaire as a result of the activities carried out in the respective voyage episodes; it was explained as effect of their job-related stress. Additionally, the seafarers were asked in free text about their most common psychophysical reactions. The questionnaire was administered in English.

During a pilot study, this questionnaire was tested and adapted with a sample of 200 seafarers. Furthermore, the study participants recorded their working, leisure and sleeping times in the recorded examination period as accurately as possible to the minute and over a period of 3 consecutive days that included all three voyage episodes.

At the same time, a continuous objective survey of strain was carried out on board by using the SenseWear armband monitor and the RS 800 Polar watch during the entire survey period. The SenseWear armband provided an objective measure of the physical activity based on the wearer's steps and the sailor’s calorie expenditure. The armband monitor has already been tested and successfully used as an activity measuring system in numerous studies [19–21]; it has proved to be superior in comparison to other commercially available activity monitors [22].

The Polar watch RS 800 Multi provided data on heart rate and heart rate variability (standard deviation of normal to normal R-R intervals (SDNN), where R is a point corresponding to the peak of the QRS complex of the ECG wave; and RR is the interval between successive Rs). The Polar watch has also already been applied in various studies as a suitable measure to assess the mentioned cardiac parameters [23, 24]. The average heart rate was determined over a 24-hour and a 4-hour period (adapted to the working shift of the nautical officers). In addition, the percentage of the working pulse above the continuous power limit was calculated. On the basis of the recommendations of the German Employers’ Liability Insurance Association for the Construction Industry (Berufsgenossenschaft Bau), this limit was defined as a heart rate of 110 beats per minute (min^-1^) for pragmatic reasons; it is assumed that the largest part of the employable population will not be overstrained if this limit is maintained—based on a working shift duration of 8 hours [25].

Additionally, in a pilot study 4 examiners wore the SenseWear armband monitor and the RS 800 Polar watch for 3 days during their stay ashore and on board and recorded their physical activity. They observed high validity for both devices. Overall, the physical activity, heart rate and calorie expenditure of 236 sailors were measured completely over an average 2.8-day study period (between 2.5 and 6 days).

### Data analysis

Continuous, normally distributed variables were expressed as mean (± standard error (SE)) and skewed variables as median (from minimum to maximum). Repeated measures ANOVA-test and subsequently the t-test with Bonferroni correction were used for within-subject analyses. Furthermore, the mixed model with different variances proved most suitable. In the mixed models, the sailors were modeled as a random effect. The differences between certain means were calculated and the estimated marginal means given as estimates for the predicted means of the cells in the model. In a following step, the adjustment variable "age" was added. Furthermore, an additional adjustment for the parameters "subjective stress on board (caused by physical or mental stressors or by vibration, noise, ships’ movements)" as well as the "objective recording of the sea condition from the ship's journal" was tested. All reported p-values were two-tailed and a p-value <0.05 was considered statistically significant.

## Results

### Voyage episodes during the 22 ship voyages

Table 1 shows that the voyages in the present study consisted of 321 different voyage episodes, with 90 ports called at, often in conjunction with a river passage. Overall, the voyage episodes of port stay and sea passage each lasted about 40% of the maximum time share of the voyages, whereas the river passage only took up a time portion of about 17%.

**Table 1:** Overview of the distribution of the three voyage episodes during all 22 voyages.

	Total	Port stay	River passage	Sea passage
**Number of voyage episodes,** *n*	321	90	162	69
**Duration of the voyage episodes,** *days* : *hours*	179 : 7	78 : 4	31 : 1	70 : 2
**Share of voyage episode duration over the entire period of the study,** *%*	100%	43.6%	17.3%	39.1%

### Subjective stress and strain depending on the voyage episode

At 37.8%, the overall seafarer sample rated the port stay as the voyage episode with the highest stress level, followed by the river passage (24.8%) and then the sea passage (13.0%). Accordingly, there were differences in the subjective assessment of the physical and mental strain as a result of the condition of the operation; physical strain was experienced significantly more intensively amongst seafarers during port stay (Table 2). Mental strain also tended to be more pronounced during this episode, followed by river passage and, most rarely, during the sea passage. The most common psychophysical reactions were “fatigue” and “a fast heartbeat”.

**Table 2:** Seafarers' exposure according to voyage episode.

	Subjective strain during the voyage episode			
	*Port stay*	*River passage*	*Sea passage*	p^a^
***Psychophysical strain***, *score*^b^ *(SD)*				
*Physical*	0.23 (0.53)	0.14 (0.39)	0.14 (0.41)	**0.003**
*Mental*	0.25 (0.55)	0.18 (0.46)	0.20 (0.49)	0.107
***Strain caused by physical stressors***, *score*^b^ *(SD)*				
*Noise*	0.23 (0.50)	0.22 (0.47)	0.19 (0.46)	0.457
*Vibration*	0.14 (0.41)	0.28 (0.54)	0.26 (0.52)	**<0.001**
*Heat*	0.07 (0.30)	0.05 (0.23)	0.05 (0.25)	0.392
*Ships‘ movement*	0.08 (0.31)	0.14 (0.44)	0.28 (0.52)	**<0.001**

^a^repeated measures ANOVA-test

^b^scale from 0 (= not at all strainful) to 2 (= very strainful)

Furthermore, it was found that noise and vibration (with the exception of the ships’ movements while traveling at sea) were regarded as the main stressors in the total sample. In contrast, the effect of heat played a much smaller role. Additionally, seafarers did not perceive significant differences in noise or heat exposure during the three voyage episodes (Table 2). Ships’ movements were expected to occur for all episodes, but mainly during sea passages. During port stay, all occupational groups experienced significantly less strain through vibration than during the other voyage episodes. Further t-tests performed between two different voyage episodes proved that the subjective physical strain was significantly higher during port stay compared to river passage or sea passage (p = 0.015 and 0.031), whereas strain due to vibration and ships’ movements was significantly less often rated during the vessel’s stays in port (each p< 0.001).

### Daily record

With regard to the distribution of working hours recorded in the daily log, significant differences were found between the various voyage episodes; the working time during the sea passage was on average shorter than during port stay or the river passage (p< 0.001) (Table 3). As a result, seafarers had more time for recreational activities and sleep during the sea passage. During the river passage, the time outside the work commitments was used to a greater extent for sleeping.

**Table 3:** Average time (h per day and % of voyage) with different activity levels.

	Voyage episode		
	*Port stay (43*.*6%)*	*River passage (17*.*3%)*	*Sea passage (39*.*1%)*
**Activity level,** *h per day (% of the voyage episode)*			
Working hours	11.1 (46.5%)	11.7 (48.6%)	8.2 (34.0%)
Leisure time	6.0 (25.0%)	4.8 (19.9%)	7.0 (29.0%)
Sleeping time	6.9 (28.5%)	7.6 (31.5%)	8.9 (37.0%)
**Number of steps,** *(n (SD))*	11,316 (497)	11,710 (321)	7,607 (436)

SD: standard deviation

The average number of steps per minute also differed between the voyage episodes (p< 0.001)—with higher activity especially during port visits and the river passages.

### Biometric methods

The total energy expenditure differed between the voyage episodes and was significantly lower during the sea passage (Table 4). When the working time was considered exclusively, there was a tendency towards reduced work energy expenditure also during the sea passage. The energy expenditure was very similar during port stay and the river passage in both the total and the work energy balance with just over 3,300 kcal and 700 kcal respectively.

**Table 4:** Objective strain parameters depending on the voyage episode.

	Voyage episode			
	*Port stay*	*River passage*	*Sea passage*	p^a^
**Biometric data according to armband monitor,** estimated marginal means (SD)				
- TEE^b^ in kJ	14,011 (581)	14,063 (573)	12,060 (587)	**0.028**^d^
- TEE^b^ in kcal	3,348 (139)	3,361 (137)	2,882 (140)	
- WEE^c^ in kJ	3,016 (459)	3,009 (452)	2,768 (463)	0.079^d^
- WEE^c^ in kcal	721 (110)	719 (108)	662 (111)	
**Biometric data according to Polar watch,** estimated marginal means (SD)				
- Heart rate^e^, *min*^*-1*^	83.1 (12.1)	82.8 (11.9)	78.0 (12.1)	**0.008**
% Heart rate > 110 beats min^-1^	12.5%	12.4%	12.1%	0.519
- SDNN in ms	15.9 (1.2)	14.8 (1.1)	15.6 (1.1)	0.462

^a^Mixed Model: p-value calculation for interaction of voyage episode and rank

^b^TEE (Total energy expenditure): total energy expenditure per day

^c^WEE (Work-related energy expenditure): energy expenditure based on a four-hour working time

^d^adjusted for age and body mass index

^e^percentage of time above heart rate> 110 min^-1^ relative to a 4 h shift

In addition, the mean heart rate differed significantly between the voyage episodes (p = 0.001); in particular when traveling at sea, the crew had a significantly lower heart rate than during the other two voyage episodes (Table 4). The continuous performance limit (> 110 beats per minute with respect to a 4-hour working shift) was exceeded during 12.3% of the working time, irrespective of the voyage episodes. Furthermore, there was no difference in heart rate variability between voyage episodes.

After adjustment for age, subjective stress on board (due to physical or mental demands or due to vibration, noise, and ships’ movements) as well as the objective recording of the sea condition from the ship's journal, the described associations between the voyage episodes and the biometric data remained significant.

## Discussion

During a ship’s voyage, very different demands are made on the crews during the different voyage episodes [12]. It is assumed that there is an accumulation of tasks, especially during port stay [26]. Accordingly, most of the participants in this study rated this episode as "generally the most stressful", followed by the river passage and the sea passage. This coincides with the assessment of the physical and mental strain in the present survey immediately after the examined voyage episodes and thus indicates that our onboard investigation did not take place during unusual/ extreme sea voyages. The finding that a psychophysical response had been triggered by more than one fifth of the crew members during port stay indicates a high stress level while working on board. Accordingly, the continuous performance limit for heart rate was exceeded during an average of 12.3% of the recorded working time.

In the overall seafarer sample, physical strain was experienced significantly more intensively during port stay. This high burden in the port is due, among other things, to the physically demanding loading and unloading operation, the need for a watchkeeper standby, extensive repairs while the engine is not running as well as the intake of provisions and fuel [27]. The psychosocial stress was also more pronounced during this voyage episode than during the river or sea passage. This means, for example, that the considerable administrative burden resulting from the differing requirements of the shipping company, charterer and the domestic and foreign authorities during port stay also place considerable stress on the ship's command [28].

In light of this high stress level, preventive medical care to relieve the crews, in particular during port stay, would be beneficial. Most of the work requirements listed above can only be met in port and cannot be dealt with during the other voyage episodes [12]. Therefore, good time and planning management is necessary to already prepare some of the next port activities in the previous voyage episodes. For example, the stability calculation for the forthcoming loading of the ship with containers can already be made during the preceding sea passage. However, this also requires good and, above all, timely communication with the shipping company and port logistics (for example, regarding the number and weight of expected containers) [29, 30]. Better coordination with the port authorities (for example, official contact with the captain, taking into account his sleeping times) would be desirable; however, that is unfortunately often not realistic due to dynamic port operations. In addition, the ship’s crew is under time pressure in port because ship handling has to be quick, since stays in port are associated with high costs for the shipping company.

The slightly lower heart rate variability during the river passage can be interpreted as increased mental stress in this voyage episode. In line with this, it is assumed that in the face of increased maneuvering activity with growing vessel sizes ("monitoring of the area"), the journey through the river, including loading and unloading maneuvers, means a higher mental stress [31, 32].

From the typical physical influences on board (noise, heat, ships’ movements or sea condition), noise and vibration were highlighted as subjective strain parameters. Ships’ movements occur, as expected, mainly during the river or sea passage; but ships’ movements or vibration can also occur alongside the quay as a result of the operation of the ship’s engine or when heavy containers are set down on the ship’s deck; this obviously triggers stress. This study reveals low subjective strain due to physical impacts so that these stressors do not seem to disturb the seafarers noticeably [1, 2]. Moreover, an adjustment for the subjective stressors on board (through physical or mental requirements or vibration, noise, ships’ movements) and the sea condition proves that these parameters have no effect on the objective strain (heart rate (variability), energy expenditure or number of steps).

In view of the limited possibilities of intervention during port operation and also the river passage, it is necessary to strengthen the psychophysical resources of the crew, in particular during the obviously subjectively and objectively less demanding sea passage [33]. This study documents for the first time that ships operating in the coastal feeder service spend almost 60% of the total travel time in the port or in the area (river or canal). Therefore, the possible intervention framework is mainly based on the sea passage with a time share of approximately 40% of the total voyage. It is evident from the significantly lower working time and higher leisure and sleeping time while traveling at sea that this is partly already practiced.

Correspondingly, the average heart rate of seafarers during sea passages is significantly lower than during the other two voyage episodes. In this study, heart rate was not assessed under standardized conditions, hence the increased physical activity whilst in port may well have led to an increased heart rate. However, these objective data correspond with the seafarers‘ subjective assessments concerning their strain on board.

The total energy expenditure during the sea passage was significantly lower, but the work energy expenditure was not (significantly) lower. This is an expression of the fact that the work intensity during the sea passage is clearly only slightly lower, but there is a much higher proportion of leisure time, which is obviously used by the crews for regeneration, leading to a lower heart rate.

Concerning intervention strategies on board, it should be highlighted that the approach to only “strengthen the psychophysical resources of the crew” is a secondary measure of intervention (support in coping with stressors). Slišković et al. (2017) [34] emphasize that more intervention strategies should focus on moderating the main job-related stressors (primary measures) to reduce mental health risks among seafarers. Psychosocial interventions that focus only on the individual level while eschewing the broader work environment in which those individuals are expected to perform, may lead to further frustration and disengagement [35]. A limitation of this study is that only 22 container vessels could be examined in this time-consuming field study on board. The stress and strain of seafarers on other vessel types (e.g. tankers, fishing vessels) could not be assessed either. Furthermore, this study was conducted using a cross-sectional design that did not allow for the measurement of longitudinal effects. Moreover, assessing the outcome variables of strain only subjectively limits the validity of the statements. However, this subjective assessment of the strain has been shown in previous studies as a reliable method [36, 37].

Factors contributing to stress in port are varying, depending on the shipping companies’ policies. Different ports may also give rise to different stress levels. Thus, as a further limitation, it is unclear how representative the current sample of seafarers was. In light of the very personnel-intensive approach of a maritime field study, however, no current stress survey with a comparably high scope of investigations is available. Another limitation of this study is that the seafarers rated their strain in the presence of the research staff on board; this may have increased the likelihood of socially desirable answers. This is surely a general drawback of maritime field studies, but, on the other hand, only this kind of study makes it possible to obtain reliable objective parameters of the strain on board in a controlled manner.

In view of the high stress level and the objective and subjective increased strain on seafarers, tailor-made onboard health prevention measures are needed. This study indicates that there is more time to deliver on-board psychosocial interventions when ships are at sea. In particular, care should be taken in this voyage episode to ensure adequate sleeping times. Due to the high strain of seafarers, intervention actions for the promotion and improvement of individual and socio-environmental health status are recommended for future studies. These studies should also evaluate the benefit of needs-adapted leisure activities (including attractive sport offers on board, healthy and varied diet, training on health issues and relaxation training) that may contribute to an increase in the sailors’ resilience.

## Supporting information

S1 Questionnaire Questionnaire voyage episode. (DOCX)
