# Supplementary material for: Stress and strain among merchant seafarers differs across the three voyage episodes of port stay, river passage and sea passage
Source: PLoS One. 2019 Jun 4;14(6):e0217904. doi: 10.1371/journal.pone.0217904 (PMC6548393; doi:10.1371/journal.pone.0217904)
Supplement: S1 Questionnaire — (DOCX) [file pone.0217904.s001.docx]

**Questionnaire on your strain* during your last voyage episode**

***ID-Nr. __________________________***

***Date:_________________________ time:_________________________***

***Voyage episode:*** 🞏 sea passage 🞏 river/ canal passage 🞏 stay in port including mooring/ unmooring

|  | Yes, but I find it not at all  strainful* | Yes, and I find it somewhat strainful* | Yes, and I find it  very strainful |
| --- | --- | --- | --- |
| Valid for the ***last voyage episode (from ………. o’clock to ………. o’clock)***: |  |  |  |
| My workplace was characterized by |  |  |  |
| *high noise level* | 🞏 | 🞏 | 🞏 |
| *strong vibrations* | 🞏 | 🞏 | 🞏 |
| *strong ship movement* | 🞏 | 🞏 | 🞏 |
| *high temperature* | 🞏 | 🞏 | 🞏 |
| *high physical demand* | 🞏 | 🞏 | 🞏 |
| *high mental demand* | 🞏 | 🞏 | 🞏 |

*Strain is defined as a result of the activities carried out in the respective voyage episodes; it is the effect of your job-related stress.
